# Rapid diagnostic tests versus RT–PCR for Ebola virus infections: a systematic review and meta-analysis

**DOI:** 10.2471/BLT.21.287496

**Published:** 2022-06-01

**Authors:** Basilua Andre Muzembo, Kei Kitahara, Ayumu Ohno, Ngangu Patrick Ntontolo, Nlandu Roger Ngatu, Keinosuke Okamoto, Shin-Ichi Miyoshi

**Affiliations:** a Graduate School of Medicine, Dentistry and Pharmaceutical Sciences, Okayama University, 1-1-1 Tsushimanaka, Kita Ward, Okayama, 700-8530, Japan.; b Collaborative Research Center of Okayama University for Infectious Diseases in India, Kolkata, India.; c Institut Médical Evangélique, Kimpese, Democratic Republic of the Congo.; d Department of Public Health, Kagawa University Faculty of Medicine, Miki, Japan.

## Abstract

**Objective:**

To evaluate the clinical accuracy of rapid diagnostic tests for the detection of Ebola virus.

**Methods:**

We searched MEDLINE®, Embase® and Web of Science for articles published between 1976 and October 2021 reporting on clinical studies assessing the performance of Ebola virus rapid diagnostic tests compared with reverse transcription polymerase chain reaction (RT–PCR). We assessed study quality using the QUADAS-2 criteria. To estimate the pooled sensitivity and specificity of these rapid diagnostic tests, we used a bivariate random-effects meta-analysis.

**Findings:**

Our search identified 113 unique studies, of which nine met the inclusion criteria. The studies were conducted in the Democratic Republic of the Congo, Guinea, Liberia and Sierra Leone and they evaluated 12 rapid diagnostic tests. We included eight studies in the meta-analysis. The pooled sensitivity and specificity of the rapid tests were 86% (95% confidence interval, CI: 80–91) and 95% (95% CI: 91–97), respectively. However, pooled sensitivity decreased to 83% (95% CI: 77–88) after removing outliers. Pooled sensitivity increased to 90% (95% CI: 82–94) when analysis was restricted to studies using the RT–PCR from altona Diagnostics as gold standard. Pooled sensitivity increased to 99% (95% CI: 67–100) when the analysis was restricted to studies using whole or capillary blood specimens.

**Conclusion:**

The included rapid diagnostic tests did not detect all the Ebola virus disease cases. While the sensitivity and specificity of these tests are moderate, they are still valuable tools, especially useful for triage and detecting Ebola virus in remote areas.

## Résumé

**Objectif:**

Évaluer la précision clinique des tests de diagnostic rapide pour dépister le virus Ebola.

**Méthodes:**

Nous avons exploré les bases de données MEDLINE®, Embase® et Web of Science à la recherche d'articles, publiés entre 1976 et octobre 2021, qui évoquaient des études cliniques mesurant les performances des tests de diagnostic rapide du virus Ebola comparés à une réaction en chaîne par polymérase après transcription inverse (RT-PCR). Nous avons déterminé la qualité de ces études à l'aide des critères QUADAS-2. Enfin, pour estimer la sensibilité et la spécificité regroupées de ces tests de diagnostic rapide, nous avons eu recours à une méta-analyse bivariée à effets aléatoires.

**Résultats:**

Nos recherches nous ont permis d'identifier 113 études uniques, dont neuf correspondaient aux critères d'inclusion. Ces études avaient été menées en Guinée, au Libéria, en République démocratique du Congo et en Sierra Leone, et portaient sur douze tests de diagnostic rapide. Nous avons inclus huit études dans notre méta-analyse. La sensibilité et la spécificité combinées des tests rapides s'élevaient respectivement à 86% (intervalle de confiance de 95%, IC: 80–91) et 95% (IC de 95%: 91–97). Néanmoins, la sensibilité combinée baissait à 83% (IC de 95%: 77–88) après retrait des valeurs aberrantes. Lorsque l'analyse se limitait aux études utilisant la RT-PCR d'altona Diagnostics en guise de référence, la sensibilité combinée augmentait jusqu'à 90% (IC de 95%: 82–94). Elle atteignait même 99% (IC de 95%: 67–100) quand l'analyse se limitait aux études basées sur des échantillons de sang total ou capillaire.

**Conclusion:**

Les tests de diagnostic rapide pris en compte ne détectaient pas tous les cas de maladie à virus Ebola. Néanmoins, en dépit d'une sensibilité et d'une spécificité modérées, ils demeurent de précieux outils, en particulier pour le triage et le dépistage du virus Ebola dans les zones reculées.

## Resumen

**Objetivo:**

Evaluar la precisión clínica de las pruebas diagnósticas rápidas para la detección del virus del Ébola.

**Métodos:**

Se realizaron búsquedas en MEDLINE®, Embase® y Web of Science de artículos publicados entre 1976 y octubre de 2021 que informaran sobre estudios clínicos en los que se evaluara el rendimiento de las pruebas diagnósticas rápidas para detectar el virus del Ébola en comparación con la reacción en cadena de la polimerasa con retrotranscripción (RT-PCR). Se evaluó la calidad de los estudios mediante los criterios QUADAS-2. Para estimar la sensibilidad y la especificidad combinadas de estas pruebas diagnósticas rápidas, se utilizó un metanálisis bivariante de efectos aleatorios.

**Resultados:**

La búsqueda identificó 113 estudios singulares, de los que nueve cumplían los criterios de inclusión. Los estudios se realizaron en la República Democrática del Congo, Guinea, Liberia y Sierra Leona y evaluaron doce pruebas diagnósticas rápidas. Se incluyeron ocho estudios en el metanálisis. La sensibilidad y la especificidad agrupadas de las pruebas rápidas fueron del 86 % (intervalo de confianza del 95 %, IC: 80-91) y del 95 % (IC del 95 %: 91-97), respectivamente. Sin embargo, la sensibilidad agrupada disminuyó al 83 % (IC del 95 %: 77-88) tras eliminar los valores atípicos. La sensibilidad agrupada aumentó al 90 % (IC del 95 %: 82-94) cuando el análisis se restringió a los estudios que utilizaban la RT-PCR de altona Diagnostics como criterio de referencia. La sensibilidad agrupada aumentó al 99 % (IC del 95 %: 67-100) cuando el análisis se restringió a los estudios que utilizaban muestras de sangre total o capilar.

**Conclusión:**

Las pruebas diagnósticas rápidas incluidas no detectaron todos los casos de enfermedad por el virus del Ébola. Aunque la sensibilidad y la especificidad de estas pruebas son moderadas, siguen siendo herramientas valiosas, sobre todo útiles para el triaje y la detección del virus del Ébola en zonas remotas.

## ملخص

**الغرض:**

تقييم الدقة السريرية لاختبارات التشخيص السريع للكشف عن فيروس الإيبولا.

**الطريقة:**

قمنا بالبحث في **MEDLINE®‎ ** و **Embase®‎ ** و **Web of Science ** عن المقالات المنشورة بين 1976 وأكتوبر/تشرين أول 2021 التي تشير إلى الدراسات السريرية، التي تقيّم أداء اختبارات التشخيص السريع لفيروس الإيبولا، بالمقارنة مع النسخ العكسي لتفاعل البوليميراز المتسلسل ( **RT–PCR **). قمنا بتقييم جودة الدراسة باستخدام معايير **QUADAS-2 **. لتقدير الحساسية والخصوصية المجمعة لهذه الاختبارات التشخيصية السريعة، قمنا باستخدام التحليل التلوي للتأثيرات العشوائية المزدوجة.

**النتائج:**

حدد بحثنا 113 دراسة فريدة، تسعة منها حققت معايير الاشتمال. تم إجراء الدراسات في جمهورية الكونغو الديمقراطية وغينيا وليبيريا وسيراليون، وقامت بتقييم اثني عشر اختبارًا تشخيصيًا سريعًا. قمنا بتضمين ثماني دراسات في التحليل التلوي. كانت الحساسية والخصوصية المجمعة للاختبارات السريعة 86% (بفاصل ثقة مقداره 95%: 80 إلى 91) و95% (بفاصل ثقة مقداره 95%: 91 إلى 97)، على الترتيب. ومع ذلك، انخفضت الحساسية المجمعة إلى 83% (بفاصل ثقة مقداره 95%: 77 إلى 88) بعد إزالة القيم المتطرفة. انخفضت الحساسية المجمعة إلى 90% (بفاصل ثقة مقداره 95%: 82 إلى 94) عندما اقتصر التحليل على الدراسات التي تستخدم **RT-PCR ** من **altona Diagnostics ** باعتبارها معيار ذهبي. انخفضت الحساسية المجمعة إلى 99% (بفاصل ثقة مقداره 95%: 67 إلى 100) عندما اقتصر التحليل على الدراسات التي تستخدم عينات دم كاملة أو شعيرية.

**الاستنتاج:**

لم تكتشف الاختبارات التشخيصية السريعة المُضمّنة جميع حالات الإصابة بمرض فيروس الإيبولا. في حين أن حساسية وخصوصية هذه الاختبارات معتدلة، إلا أنها لا تزال أدوات ذات قيمة كبيرة، ومفيدة بشكل خاص لفرز واكتشاف فيروس الإيبولا في المناطق النائية.

## 摘要

**目的:**

评估埃博拉病毒快速诊断检测法的临床准确性。

**方法:**

我们搜索了 MEDLINE®、Embase® 和 Web of Science 上于 1976 年至 2021 年 10 月期间发表的文章，这些文章报道了埃博拉病毒快速诊断检测与实时逆转录聚合酶链反应 (RT–PCR) 相比的效果评估。我们采用 QUADAS-2 标准评估了研究的质量，并且采用双变量随机效应元分析来评估这些快速诊断检测的综合敏感性和特异性。

**结果:**

我们搜索了 113 项研究，其中有九项符合纳入标准。这些研究是在刚果民主共和国、几内亚、利比里亚和塞拉利昂开展的，研究评估了十二种快速诊断检测方法。我们在元分析中纳入了八项研究。快速检测的综合敏感性和特异性分别为 86%（95% 置信区间，CI: 80–91）和 95% (95% CI: 91–97)。但是，在去除离群值之后，综合敏感性降至 83% (95% CI: 77–88)。当分析限定于将采用来自 altona Diagnostics 公司的 RT–PCR 检测作为黄金标准的研究时，综合敏感性增至 90% (95% CI: 82–94)。当分析限定于采用全血标本或末梢采血标本的研究时，综合敏感性增至 99% (95% CI: 67–100)。

**结论:**

纳入的快速诊断检测并未检测出所有埃博拉病毒病例。尽管这些检测在敏感性和特异性方面表现一般，但仍然是有价值的工具，尤其适用于偏远地区的埃博拉病毒分诊和检测。

## Резюме

**Цель:**

Оценить клиническую точность экспресс-тестов для диагностики вируса Эбола.

**Методы:**

Авторы провели поиск в базах данных MEDLINE®, Embase® и Web of Science статей, опубликованных в период с 1976 г. по октябрь 2021 г., в которых сообщалось о клинических исследованиях, оценивающих эффективность экспресс-тестов для диагностики вируса Эбола по сравнению с полимеразной цепной реакцией с обратной транскрипцией (ОТ-ПЦР). Авторы оценивали качество исследования с использованием критериев QUADAS-2. Для оценки совокупной чувствительности и специфичности диагностических экспресс-тестов авторы использовали двумерный метаанализ случайных эффектов.

**Результаты:**

Поиск выявил 113 уникальных исследований, девять из которых соответствовали критериям включения. Исследования проводились в Гвинее, Демократической Республике Конго, Либерии и Сьерра-Леоне, в которых оценивались двенадцать диагностических экспресс-тестов. Авторы включили восемь исследований в метаанализ. Совокупная чувствительность и специфичность экспресс-тестов составила 86% (95%-й ДИ: 80–91) и 95% (95%-й ДИ: 91–97) соответственно. Однако совокупная чувствительность снизилась до 83% (95%-й ДИ: 77–88) после удаления выбросов. Совокупная чувствительность увеличилась до 90% (95%-й ДИ: 82–94), когда анализ был ограничен исследованиями с использованием ОТ-ПЦР от altona Diagnostics в качестве главного стандарта. Совокупная чувствительность увеличилась до 99% (95%-й ДИ: 67–100), когда анализ был ограничен исследованиями с использованием образцов цельной или капиллярной крови.

**Вывод:**

Включенные диагностические экспресс-тесты не выявили всех случаев болезни, вызванной вирусом Эбола. Хотя чувствительность и специфичность этих тестов умеренные, они по-прежнему являются ценными инструментами, особенно полезными для сортировки больных и диагностики вируса Эбола в удаленных районах.

## Introduction

Ebola virus disease was first discovered in 1976 in the Democratic Republic of the Congo and South Sudan.^1^ This highly pathogenic disease is often fatal in humans; in past outbreaks the case fatality rate ranged from 25% (37/149) to 90% (128/143).^1^ At the initial stages of the disease, symptoms include fever, vomiting, diarrhoea, anorexia and fatigue.^2^ Diagnosing the disease on these symptoms alone is challenging, because they are similar to common endemic diseases present in Africa, such as typhoid fever, malaria and yellow fever.^3^^,^^4^

To confirm the diagnosis of Ebola virus disease, a positive result from a reverse transcription polymerase chain reaction (RT–PCR) test is required.^5^ However, lateral flow assays (that is, rapid diagnostic tests) are valuable tools to limit the spread of the disease since their fast turnaround time has the potential to trigger early outbreak alerts. For instance, researchers estimated that if a combination of rapid diagnostic tests and RT–PCR assays had been available during the 2013–2016 Ebola virus disease outbreak, the number of infections would have been up to a third less in Sierra Leone.^6^

Most Ebola outbreaks begin in remote or rural areas^1^ with limited hospital availability and trained clinicians. Laboratory equipment needed for diagnosis and trained equipment users are rarely available; it can take hours or days to get the RT–PCR results.^7^ If rapid diagnostic tests for the disease were readily available in high-risk outbreak areas, lives could be saved since the time between virus introduction into a community and implementation of countermeasures could be decreased.^8^

According to the World Health Organization (WHO), rapid diagnostic tests for Ebola virus should have a desired clinical sensitivity of > 98% and an acceptable clinical sensitivity of more than 95%.^9^ Since 1976, many Ebola virus rapid diagnostic tests have been developed, but researchers have not yet thoroughly assessed the evidence of their performance in clinical samples. The few rapid diagnostic tests that have been assessed in field conditions demonstrated uncertainty and variability in performance.^6^ We therefore conducted a meta-analysis to increase the evidence base of current rapid diagnostic tests detecting Ebola virus in suspected cases.

## Methods

We conducted a systematic review and meta-analysis of studies that assessed the performance of rapid diagnostic tests for Ebola virus compared with RT–PCR. We followed the Preferred Reporting Items for a Systematic Review and Meta-analysis of Diagnostic Test Accuracy Studies.^10^ This review is registered with the International Prospective Register of Systematic Reviews (CRD42021278280).

We searched MEDLINE®, Embase® and Web of Science for articles published from 1976 to 7 October 2021. Search terms used are available in Box 1. We applied no language restrictions during the search. We also hand-searched the articles included in the reference lists of relevant studies, related key reviews and a book chapter on Ebola rapid diagnostic tests.^8^ The studies that we retrieved were exported to EndNote software X9 (Clarivate, Philadelphia, United States of America) and from there, we removed duplicated studies.

Box 1 **Search terms and keywords used to identify studies on diagnostic accuracy of rapid tests for Ebola virus disease**

For Ebola virus disease we used the keywords:“Ebola virus disease” [MeSH Terms] OR “Ebola virus” [All fields] OR “Ebola” [All fields] OR “hemorrhagic fever” [All fields].

We combined these keywords with: “rapid diagnostic test” [MeSH Terms] OR “Rapid test” [All fields] OR “rapid assay” [All fields] OR “EBOV lateral flow assay” [All fields].

We further narrowed searches by including the following names for index test: “ReEBOV,” “QuickNaviTM-Ebola,” “eZYSCREEN,” “DSTL EVD lateral flow assay,” “OraQuick Ebola rapid antigen test kit,” “SD Q Line Ebola Zaire Ag” and “NMRC EBOV LFI.”

Two authors screened all the titles and abstracts identified through the search, and reviewed the full text of potentially relevant articles against the inclusion and exclusion criteria (Box 2). Each researcher was blind to the selection of the other researcher. We recorded reasons for excluding articles; disagreements were discussed and arbitrated by consensus.

Box 2 **Inclusion and exclusion criteria used to identify studies on diagnostic accuracy of rapid tests for Ebola virus disease**

**Inclusion criteria:**

- Patients: individuals with suspected Ebola virus disease;
- Index test: classic and non-classic lateral flow assays carried out in any specimen to diagnose Ebola virus disease;
- Reference standard: the study evaluates rapid diagnostic tests for Ebola virus against RT–PCR;
- Outcomes: the study reports sensitivity and specificity of rapid diagnostic tests for Ebola virus or contains sufficient data to calculate sensitivity and specificity, by recreating the 2 × 2 diagnostic table.

**Exclusion criteria:**

- Rapid PCR-based Ebola virus tests;
- Review articles, editorials and non-clinical studies.

RT–PCR: reverse transcription polymerase chain reaction.

### Data extraction

Two investigators independently extracted data from individual studies. We then compared extracted data and any disagreements were resolved through discussion.

Prior to data extraction, we designed a standardized data extraction form. Extracted data included: setting, study period, sample size, type of specimen, index test, reference standard and reported conflicts of interest. We also extracted reported sensitivity and specificity, raw data on true positives, false positives, false negatives and true negatives to recreate two-by-two tables.

### Methodological assessment

Two authors independently evaluated the methodological quality of the included studies using the Quality Assessment of Diagnostic Accuracy Studies 2 (QUADAS-2) tool.^11^ We resolved any disagreements through consensus and further article reading.

### Data synthesis and analysis

All statistical analyses were performed using Stata (version 16, StataCorp LP, College Station, USA). We applied a bivariate-effect model to calculate the pooled sensitivity, specificity, positive and negative likelihood ratios and the diagnostic odds ratio (OR). We generated forest plots of the sensitivity and specificity of each data point.

We conducted meta-analysis using the bivariate-effect models because of the heterogeneity expected between studies assessing diagnostic test accuracy.^12^ We also applied the bivariate-effect model to generate plot hierarchical summary receiver-operating characteristic curves.

Heterogeneity was assessed by visual inspection of forest plots of the sensitivity and specificity and the shape of the hierarchical summary receiver-operating characteristic curves.^13^

In our subgroup analysis, we stratified studies into seven subgroups based on: specimen type (serum alone, plasma alone, serum and plasma combined, whole blood and capillary blood combined); reference standard (RealStar Filovirus Screen RT–PCR Kit 1.0 [altona diagnostics GmbH, Hamburg, Germany, hereafter altona] alone and other RT–PCR tests such as Trombley assay);^14^ and exclusion of outliers (studies reporting that the rapid diagnostic test is 100% sensitive or specific). We also performed a subgroup analysis using only the ReEBOV™ Antigen Rapid Test kit (Corgenix Inc., Broomfield, USA; hereafter ReEBOV™) because it had enough data points to be pooled separately.

## Results

**Fig. 1:**
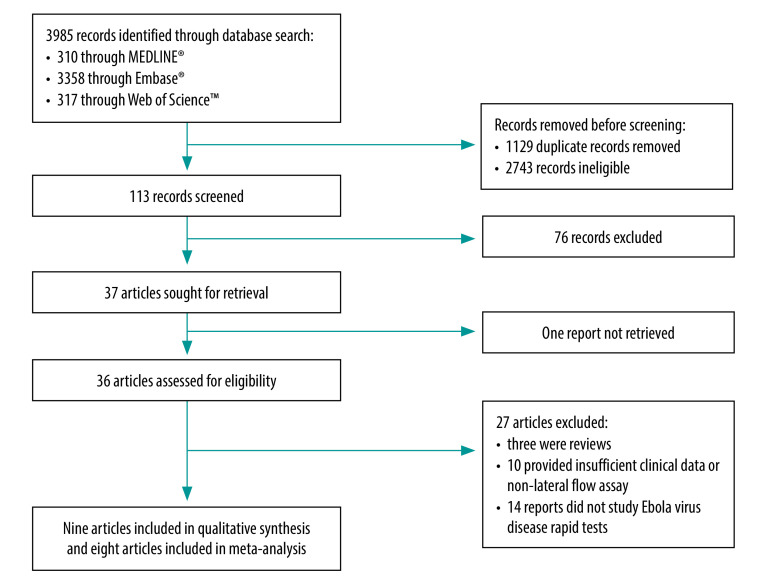
Flowchart of the selection of articles included in study on the diagnostic accuracy of rapid tests for Ebola virus disease

The searches yielded 113 studies for screening after we removed duplicates (Fig. 1). Of the 36 full-text studies that we evaluated for eligibility, nine studies met the inclusion criteria.^15^^–^^23^ Of these, eight were eligible for the meta-analysis.^15^^–^^19^^,^^21^^–^^23^ We excluded 10 studies because they were non-lateral flow assays, we were unable to reconstitute the two-by-two diagnostic table or had scarce evidence on clinical performance (e.g. studies showing only analytical performance of the rapid diagnostic test).^24^^–^^33^

### Study characteristics

The included studies were cross-sectional studies published in English^15^^–^^18^^,^^20^^–^^23^ and French^19^ between 2015 and 2020. Sample size ranged from 105 to 928 tests performed. The studies were conducted in four countries: the Democratic Republic of the Congo,^16^ Guinea,^15^^,^^19^ Liberia^20^ and Sierra Leone.^17^^,^^18^^,^^21^^–^^23^

Specimens were from either Ebola-suspected patients, those hospitalized at an Ebola treatment centre or from deceased people. One study tested postmortem oral swab specimens;^20^ all the studies tested blood specimens: capillary blood,^22^^,^^23^ whole blood,^16^^,^^17^^,^^19^ plasma^15^^,^^17^^,^^18^^,^^20^^–^^22^ and serum.^15^^,^^19^ Haemolyzed specimens were also included.^17^ Four studies clearly stated that rapid tests were performed on stored serum^15^ or stored plasma samples.^17^^,^^18^^,^^21^ Further details on study characteristics are shown in Table 1. Five studies indicated participants’ age (available in the data repository).^34^

**Table 1:** Studies included in the systematic review and meta-analysis on the diagnostic accuracy of rapid tests for Ebola virus disease

Study	Country	Sample size, no.	Study design	Reference standard^a^	Specimen	Year of specimen collection	Industry funded	Index test	No. of samples					% (95% CI)		Prevalence, %
									True positive	False positive	False negative	True negative		Reported sensitivity	Reported specificity	
Moran et al., 2020^15^	Guinea	205	Cross-sectional	RT–PCR (altona)	Stored serum	2014–2015	No	Fever Panel Antigen System	98	9	11	87		90 (83–95)	91 (83–96)	53
								Ebola Antigen System	84	8	25	88		77 (68–84)	92 (84–96)	53
								Ebola–Malaria Antigen duplex system	85	4	24	92		78 (69–85)	96 (89–99)	53
Makiala et al., 2019^16^	Democratic Republic of the Congo	928	Cross-sectional	GeneXpert® Ebola	Whole blood	2018	No	QuickNavi^TM^-Ebola	68	2	12	846		85 (75–92)	100 (99–100)	9
Wonderlyet al., 2019^17^	Sierra Leone	428	Cross-sectional	RT–PCR (altona)	Whole blood	2015	No, but tests donated	DEDIATEST Ebola	0	0	0	98		–	100 (96–100)	–
				RT–PCR (altona)	Stored plasma	2015	No, but tests donated	SD Ebola Zaire Ag	109	2	20	196		85 (77–90)	99 (96–100)	–
				RT–PCR (Trombley)	Stored plasma	2015	No, but tests donated	SD Ebola Zaire Ag	110	1	46	170		71 (63–78)	99 (97–100)	–
				RT–PCR (altona)	Stored plasma	2015	No, but tests donated	ReEBOV™ Antigen Rapid Test kit	123	39	9	159		93 (88–97)	80 (74–86)	–
				RT–PCR (Trombley)	Stored plasma	2015	No, but tests donated	ReEBOV™ Antigen Rapid Test kit	133	29	23	142		85 (79–90)	83 (77–88)	–
				RT–PCR (altona)	Stored plasma	2015	No, but tests donated	One step Ebola test	125	39	2	158		98 (94–100)	80 (74–86)	–
				RT–PCR (Trombley)	Stored plasma	2015	No, but tests donated	One step Ebola test	138	26	16	144		90 (84–94)	85 (78–90)	–
				RT–PCR (altona)	Stored plasma	2015	No, but tests donated	DEDIATEST EBOLA	101	31	26	167		80 (72–86)	84 (79–89)	–
				RT–PCR (Trombley)	Stored plasma	2015	No, but tests donated	DEDIATEST EBOLA	108	24	46	147		70 (62–77)	86 (80–91)	–
Colavita et al., 2018^18^	Sierra Leone	210	Cross-sectional	qRT–PCR (altona)	Stored residual plasma samples	2014–2015	Yes	EBOLA Ag *K*-SET	93	2	12	103		89 (81–94)	98 (93–100)	50
Gallais et al., 2017^19^	Guinea	137	Cross-sectional	qRT–PCR (altona; and Weidmann)	Whole blood	2015	No	eZYSCREEN®	32	1	17	87		65 (50–78)	99 (94–100)	36
		144	Cross-sectional	qRT–PCR (altona; and Weidmann)	Serum	2015	No	eZYSCREEN®	41	0	14	89		75 (61–85)	100 (96–100)	38
Phan et al., 2016^b,^^20^	Liberia	290	Cross-sectional	rRT–PCR (Trombley)	Plasma	2014–2015	No	NMRC Ebola virus lateral flow immunoassay	–	–	–	–		88 (75–94)	98 (95–99)	–
		237	Cross-sectional	rRT–PCR (Trombley)	Postmortem oral swabs	2014–2015	No	NMRC Ebola virus lateral flow immunoassay	–	–	–	–		89 (57–98)	96 (93–98)	–
Boisen et al., 2016^21^	Sierra Leone	176	Cross-sectional	Ebola virus-specific qRT–PCR^c^	Stored plasma	2014	Yes	ReEBOV™ Antigen Rapid Test kit	72	2	44	58		62 (53–71)	97 (89–100)	53
Broadhurst et al., 2015^22^	Sierra Leone	105	Cross-sectional	RT–PCR (altona)	Plasma (for the reference test) and capillary blood (for the rapid tests)	2015	No, but tests donated	ReEBOV™ Antigen Rapid Test kit	28	6	0	71		100 (88–100)	92 (84–97)	27
		277	Cross-sectional	RT–PCR (altona)	Whole blood	2015	No, but tests donated	ReEBOV™ Antigen Rapid Test kit	45	18	0	214		100 (92–100)	92 (88–95)	16
Walker et al., 2015^23^	Sierra Leone	131	Cross-sectional	RT–PCR (altona)	Capillary blood	2015	No	Defence Science and Technology Laboratory Ebola virus disease rapid diagnostic test	15	9	107	0		100 (78–100)	92 (86–96)	12

CI: confidence interval; PCR: polymerase chain reaction; RT–PCR: reverse transcription PCR; rRT–PCR: real-time RT–PCR; qRT–PCR: quantitative RT–PCR.

^a^ altona refers to RealStar Filovirus Screen RT–PCR Kit 1.0 (altona Diagnostics GmbH, Hamburg, Germany), Trombley refers to the Trombley assay,^14^ GeneXpert® Ebola (Cepheid, Sunnyvale, United States of America) is a specific RT-PCR that targets the glycoprotein and nucleoprotein genes, Weidmann refers to the Weidmann technique (that is, a quantitative one-step RT–PCR).^19^

^b^ We were unable to accurately reconstitute the two-by-two table used to calculate specificity and sensitivity.

^c^ RT–PCR specific for Ebola (while not specifically mentioned in the article, the authors cite the Trombley assay in their methods).

### Index tests

The included studies evaluated 12 index tests: Reba™;^17^^,^^21^^,^^22^ QuickNavi™-Ebola (Denka Seiken, Tokyo, Japan);^16^ DEDIATEST EBOLA (Senova, Weimar, Germany);^17^ One step Ebola test (Intec, Xiamen, China);^17^ SD Ebola Zaire Ag (SD Biosensor, Suwon-si, Republic of Korea);^17^ EBOLA Ag *K*-SET (Coris BioConcept, Gembloux, Belgium);^18^ eZYSCREEN® (Vedalab, Alençon, France);^19^ NMRC Ebola virus lateral flow immunoassay (Naval Medical Research Center, Bethesda, USA);^20^ Defence Science and Technology Laboratory Ebola virus disease rapid diagnostic test (Defence Science and Technology Laboratory, Salisbury, United Kingdom of Great Britain and Northern Ireland);^23^ and three tests using the dual path platform from Chembio Diagnostics (Medford, USA): Fever Panel Antigen System,^15^ Ebola Antigen System^15^ and Ebola–Malaria Antigen duplex system.^15^ Further details on the tests are available from the data repository.^34^

The tests using the dual path platform are different from classic lateral flow assays as they contain a cartridge with a battery. ReEBOV™ should be stored at 2–8 °C, hence a cold chain is needed which could be a concern for use in remote field conditions.^17^^,^^22^ In addition, ReEBOV™, DEDIATEST EBOLA and SD Ebola Zaire Ag have been reported to have operational biosafety concerns when using the test.^17^ The NMRC Ebola virus lateral flow immunoassay could not yield readable results in samples containing red blood cells.^20^

All studies compared the rapid diagnostic test against RT–PCR, notably altona,^15^^,^^17^^–^^20^^,^^22^^,^^23^ Trombley assay,^17^^,^^20^ GeneXpert® Ebola (Cepheid, Sunnyvale, USA)^16^, Ebola-specific quantitative RT–PCR (while not mentioned specifically in the article, the authors cite the Trombley assay in their methods),^21^ and Weidmann technique (that is, a quantitative one-step RT–PCR).^19^

### Methodological assessment

The results of the quality assessments are available in the data repository.^34^ In the patient selection domain, 78% (7/9) of the studies had an unclear or high risk of bias, because these studies did not clearly specify random or consecutive recruitment of participants.^15^^,^^17^^,^^18^^,^^20^^–^^23^ Furthermore, some studies had suboptimal study design (two studies),^15^^,^^22^ missing information on patients’ exclusion criteria (seven studies)^15^^,^^17^^,^^18^^,^^20^^,^^21^^,^^22^^,^^23^ and the use of stored blood samples collected for other purposes (three studies).^15^^,^^17^^,^^18^ Regarding the reference standard domain, one study was judged as having a high risk of bias for incorrect use of altona’s RT–PCR kit, by modifying the manufacturer’s instructions.^22^ We judged applicability concerns to be low in the patient selection, index test and reference standard domains.

Five (56%) studies explicitly stated no conflicts of interest.^15^^,^^16^^,^^19^^,^^20^^,^^23^ In two (22%) studies, authors acknowledged having potential conflicts of interest.^18^^,^^21^ Two (22%) studies reported receiving test kits from manufacturers but did not consider it as a conflict of interest.^17^^,^^22^

### Meta-analysis

Nineteen data points covering 5332 tests performed were available to summarize the performance of rapid diagnostic tests for Ebola virus.

**Fig. 2:**
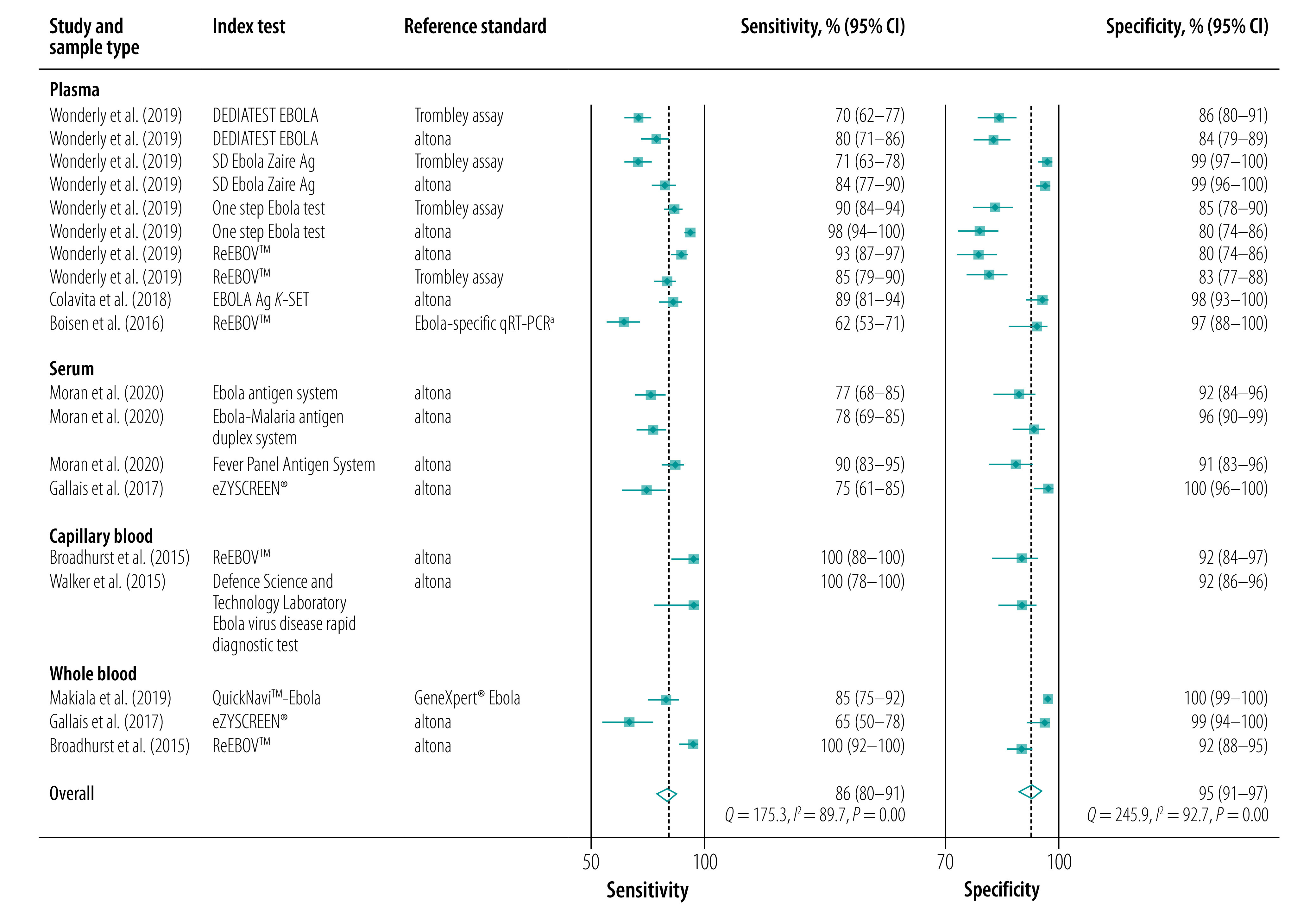
Forest plots of the sensitivities and specificities of Ebola virus rapid diagnostic tests compared with RT–PCR

**Fig. 3:**
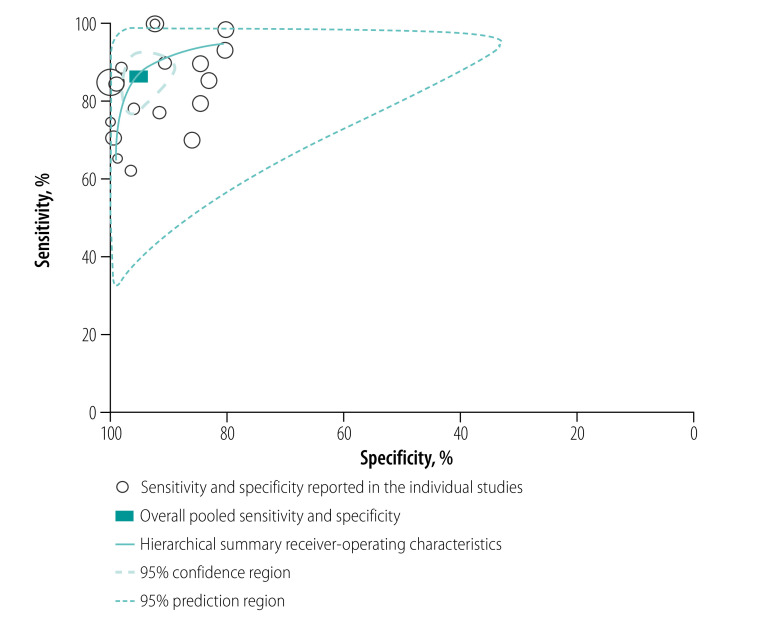
Hierarchical summary receiver-operating characteristic curves of the sensitivity and specificity of Ebola virus rapid diagnostic tests compared with RT–PCR

Table 2 shows the bivariate-effect model estimates for the pooled sensitivity, specificity, positive and negative likelihood ratios and the diagnostic OR. The pooled sensitivity and specificity were 86% (95% confidence interval, CI: 80–91) and 95% (95% CI: 91–97), respectively (Fig. 2). While sensitivity estimates varied widely from 62% to 100% across studies, the range for specificity estimates were narrower (80–100%; Fig. 3).

**Table 2:** Meta-analysis of rapid diagnostic tests for Ebola virus disease

Group and study	Data points (no. of studies)	Sample size	% (95% CI)				
			Pooled sensitivity	Pooled specificity	Positive likelihood ratio	Negative likelihood ratio	Diagnostic OR
**All studies** ^15^ ^–^ ^19^ ^,^ ^21^ ^–^ ^23^	19 (8)	5332	86 (80–91)	95 (91–97)	18.0 (9.9–32.9)	0.14 (0.10–0.21)	126 (66–240)
**Sample type**							
Serum and plasma^15^^,^^17^^–^^19^^,^^21^	14 (5)	3754	84 (77–89)	94 (89–97)	13.7 (7.4–25.5)	0.17 (0.12–0.24)	79 (42–148)
Plasma^17^^,^^18^^,^^21^	10 (3)	2995	85 (76–91)	93 (85–97)	11.9 (5.7–24.8)	0.16 (0.10–0.25)	74 (34–160)
Serum^15^^,^^19^	4 (2)	759	80 (73–86)	95 (89–98)	17.6 (7.5–41.0)	0.21 (0.15–0.28)	85 (38–190)
Whole blood and capillary blood^16^^,^^19^^,^^22^^,^^23^	5 (4)	1578	99 (67–100)	98 (91–99)	45.5 (11.3–182.7)	0.02 (0.00–0.45)	3011 (184–49 177)
**Reference standard**							
altona^a,^^15^^,^^17^^,^^18^^,^^19^^,^^22^^,^^23^	13 (6)	2925	90 (82–94)	94 (90–97)	15.9 (8.9–28.4)	0.11 (0.06–0.19)	146 (73–293)
Other standards^b,^^16^^,^^17^^,^^21^	6 (3)	2407	78 (69–86)	96 (86–99)	21.5 (5.3–87.2)	0.22 (0.15–0.32)	96 (23–408)
**Other**							
Removing outlier studies^c,^^15^^,^^16^^,^^17^^,^^18^^,^^19^^,^^21^	15 (6)	4675	83 (77–88)	95 (90–98)	16.9 (8.4–33.9)	0.17 (0.13–0.24)	97 (48–196)
ReEBOV™ Antigen Rapid Test kit^17^^,^^21^^,^^22^	5 (3)	1215	95 (70–99)	89 (83–94)	8.9 (5.3–15.1)	0.06 (0.01–0.42)	157 (18–1403)

CI: confidence interval; OR: odds ratio.

^a^ RealStar Filovirus Screen reverse transcription polymerase chain reaction Kit 1.0 (altona Diagnostics GmbH, Hamburg, Germany).

^b^ Gold standard tests were Trombley assay,^14^ GeneXpert® Ebola (Cepheid, Sunnyvale, United States of America) and RT–PCR specific for Ebola (while not specifically mentioned in the article, the authors cite the Trombley assay in their methods).^21^

^c^ We excluded studies reporting that rapid diagnostic tests were 100% sensitive or specific.

CI: confidence interval; RT–PCR: reverse transcription polymerase chain reaction.

^a^ RT–PCR specific for Ebola (while not specifically mentioned in the article, the authors cite the Trombley assay in their methods).^21^

Note: altona refers to RealStar Filovirus Screen reverse transcription polymerase chain reaction Kit 1.0 (altona Diagnostics GmbH, Hamburg, Germany).

RT–PCR: reverse transcription polymerase chain reaction.

Notes: Each open circle represents the sensitivity and specificity of each included data point (*n* = 19). The size of each circle is proportional to the sample size of the study. A curve close to the upper left-hand corner of the hierarchical summary receiver-operating characteristic curve indicates better overall performance.^35^

### Subgroup analysis

In the subgroup analyses, the pooled specificity estimates were more consistent across subgroups compared with pooled subgroup sensitivity estimates (Table 2).

#### Sample type

##### Serum and plasma

We included five studies,^15^^,^^17^^–^^19^^,^^21^ representing 3754 samples from 14 data points. Nine tests were assessed: ReEBOV™, DEDIATEST EBOLA, SD Ebola Zaire Ag, eZYSCREEN®, Ebola Antigen System, Ebola–Malaria Antigen duplex system, EBOLA Ag *K*-SET, One step Ebola test and Fever Panel Antigen System. The pooled sensitivity and specificity were 84% (95% CI: 77–89) and 94% (95% CI: 89–97), respectively.

##### Plasma

Studies assessing performance of rapid diagnostic tests on plasma samples used either ReEBOV™, DEDIATEST EBOLA, SD Ebola Zaire Ag, EBOLA Ag *K*-SET, One step Ebola test or Fever Panel Antigen System. We included three studies in this subgroup with 2995 specimens and 10 data points.^17^^,^^18^^,^^21^ The pooled sensitivity and specificity were 85% (95% CI: 76–91) and 93% (95% CI: 85–97), respectively.

##### Whole and capillary blood

Index tests used in this subgroup were eZYSCREEN®,^19^ QuickNavi-Ebola,^16^ ReEBOV™^22^ and Defence Science and Technology Laboratory Ebola virus disease rapid diagnostic test.^23^ Among five data points and 1578 specimens, the pooled sensitivity and specificity were 99% (95% CI: 67–100) and 98% (95% CI: 91–99), respectively.

#### Reference standard

##### altona

We restricted the analysis to 13 data points (2925 specimens tested) where the altona RT–PCR kit had been used as the gold standard.^15^^,^^17^^–^^19^^,^^22^^,^^23^ Index tests used in this subgroup were eZYSCREEN®, Ebola Antigen System, Ebola–Malaria Antigen duplex system, DEDIATEST EBOLA, SD Ebola Zaire Ag, EBOLA Ag *K*-SET, Fever Panel Antigen System, ReEBOV™, One step Ebola test and Defence Science and Technology Laboratory Ebola virus disease rapid diagnostic test. The pooled sensitivity increased to 90% (95% CI: 82–94) compared with the overall estimates (86%; 95% CI: 80–91), while the pooled specificity (94%; 95% CI: 90–97) was similar (95%; 95% CI: 91–97).

##### Other RT–PCR tests

We included three studies using the following reference standards: Ebola-specific qRT–PCR,^21^ Trombley^17^ and GeneXpert® Ebola assays.^16^ Index tests were ReEBOV™, DEDIATEST EBOLA, SD Zaire Ag, QuickNavi-Ebola and One step Ebola test.

Among six data points and 2407 specimens tested, the pooled sensitivity decreased to 78% (95% CI: 69–86). However, the pooled specificity (96%; 95% CI: 86–99) was comparable to the overall estimates.

#### Removing outliers

We excluded four data points that had sensitivity^22^^,^^23^ or specificity^19^ of 100%. We found that the pooled sensitivity slightly decreased to 83% (95% CI: 77–88) compared with the overall sensitivity estimates of 86% (95% CI: 80–91). However, the pooled specificity was the same as the overall specificity estimates; 95% (95% CI: 90–98) versus 95% (95% CI: 91–97).

#### ReEBOV™

**Fig. 4:**
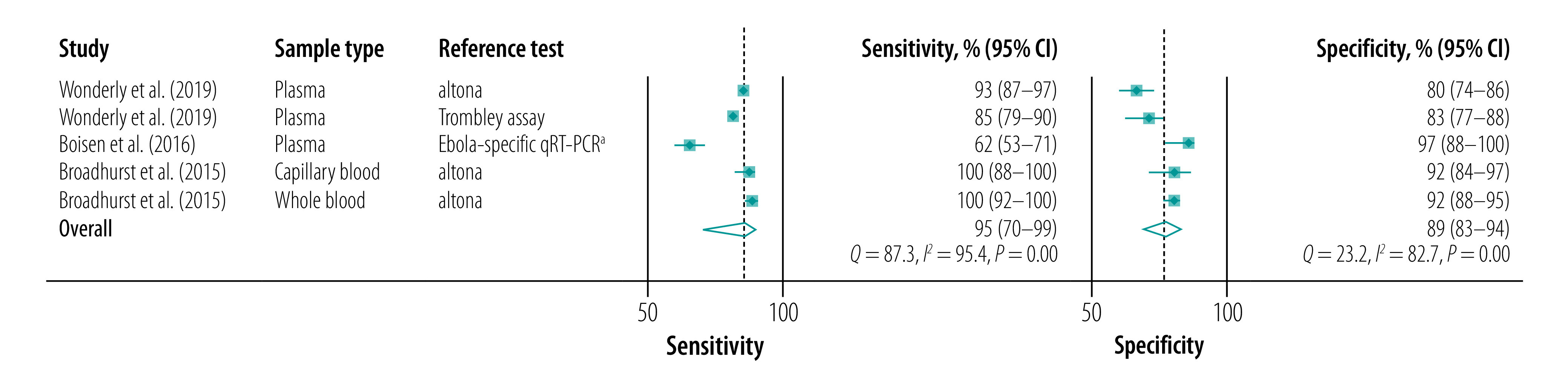
Forest plots of the sensitivities and specificities of ReEBOV™ Antigen Rapid Test kit for the detection of Ebola virus

When we considered only studies that used ReEBOV™ as index test (five data points with 1215 tests performed)^17^^,^^21^^,^^22^, the pooled sensitivity and specificity were 95% (95% CI: 70–99) and 89% (95% CI: 83–94), respectively (Fig. 4).

CI: confidence interval; RT–PCR: reverse transcription polymerase chain reaction; qRT–PCR: quantitative RT–PCR.

^a^ RT–PCR specific for Ebola (while not specifically mentioned in the article, the authors cite the Trombley assay in their methods).^21^

Note: altona refers to RealStar Filovirus Screen reverse transcription polymerase chain reaction Kit 1.0 (altona Diagnostics GmbH, Hamburg, Germany).

## Discussion

In this study, we conducted meta-analyses on clinical accuracy studies to assess the performance of rapid diagnostic tests to detect Ebola virus in individuals suspected of having the disease. Compared with the gold standard RT–PCR, the overall pooled sensitivity for rapid tests was 86%, falling short of a desired sensitivity of > 98% and an acceptable sensitivity of > 95% listed in a WHO target product profile document released during the 2013–2016 Ebola virus disease outbreak.^9^ Furthermore, we show that the overall pooled specificity was 95%, also lower than WHO’s recommended level of > 99%.^9^

While the clinical value for Ebola virus screening to contain the outbreak is indisputable, our findings suggest that better performing rapid diagnostic tests in field conditions are needed. Our results indicate that current tests miss 14% of cases, which is a considerable proportion because of the contagiousness and high mortality of Ebola virus disease. False-negative results should be minimized to the lowest level possible, since false-negative individuals might infect other people. In hospitals, false-negative patients might be treated with less precaution than positive patients and hence the likelihood of infecting health-care workers and other patients is greater. Allowing false-negative patients to wait at home for a confirmatory RT–PCR test increases the risk of infecting people in the community. In addition, false-negative patients will not be included in contact tracing, which might lead to the transmission chain being sustained. Of note is that a low viral load in the specimen could also lead to a false-negative result, even with a rapid test with high sensitivity.^36^

False positivity can have severe implications for false-positive individuals and their families since they would be subject to unnecessary quarantines. These individuals can also be exposed to the potential Ebola patients when waiting for the confirmation of the diagnosis.

When assessing in which type of sample the rapid test performed best, we found that tests made on whole or capillary blood had the highest sensitivity, specificity, likelihood ratios and diagnostic OR. Using whole or capillary blood has the advantage that blood centrifugation is not required, which would reduce the turnaround time and make the test more accessible in remote field settings. However, this subgroup analysis included only five data points, from four studies,^16^^,^^19^^,^^22^^,^^23^ and two of the data points were from a study^22^ that has been a subject of debate.^37^^–^^40^ Hence, further field studies are required to confirm results. 

Using a different gold standard also affected the results. The six data points with higher sensitivity all used altona as the gold standard.^15^^,^^17^^,^^22^^,^^23^ We also noted that pooled sensitivity was higher in studies using altona (90%) compared with other gold standards (78%). However, only six data points were available for other gold standards versus 13 for altona. It is unclear why using altona yielded a sensitivity superior to that of other gold standards. Reduced sensitivity of altona to specimens with cycle threshold values above 30 (i.e. low viral loads) have been observed.^5^^,^^41^ This reduced sensitivity of altona may affect the interpretation of our pooled estimates. Our pooled sensitivity might have been underestimated, since altona may fail to detect positive samples with low viral loads. However, we cannot rule out the possibility that studies using altona might also overestimate the sensitivity of rapid tests, since one study that used altona reported an unusual sensitivity of 100%.^22^

The review identified sufficient data points for assessing ReEBOV™ performance. This test received an emergency use authorization from the United States Food and Drug Administration (FDA) and WHO during the 2013–2016 Ebola outbreak.^26^ But, in 2018, FDA revoked this authorization when the new manufacturer (Zalgen Laboratories) that had acquired the company failed to reproduce the claimed test accuracy of ReEBOV™.^42^ The claimed sensitivity and specificity of the test were 91.8% and 84.6%, respectively.^6^ Our results are in line with the suboptimal performance: we showed a pooled sensitivity of 95% with a wide confidence interval (95% CI: 70–99), suggesting that the result is overestimated due to included outlier studies.^19^^,^^22^^,^^23^ The pooled specificity was also below WHO criteria of 99% analytical specificity.^9^

This study has some limitations. First, our meta-analysis included some studies with some methodological limitations and studies where tests’ accuracy could have been overestimated. However, this concern was addressed by conducting subgroup analyses. For instance, we assessed whether the pooled estimates differed by removing outliers in the meta-analysis. This approach did indeed change the pooled sensitivity but not its specificity. Second, synthesizing the included studies in one pooled estimate of sensitivity and specificity could be inaccurate as there was substantial variation in the used cut-off for RT–PCR cycle threshold (data repository),^34^ which limits comparability of rapid tests. These issues are illustrated by pooling only data from ReEBOV™ studies. The results showed a suboptimum pooled sensitivity with a great uncertainty.

Third, some studies used stored blood samples. Using frozen blood samples that were processed through freeze–thaw cycles may have compromised the tests’ sensitivity. Nevertheless, this explanation is unlikely since a newly published study conducted on patients also demonstrated that the sensitivity of three rapid tests (QuickNavi-Ebola, OraQuick Ebola Rapid Antigen Test (OraSure Technologies, Bethlehem, USA) and EBOLA Ag *K*-SET rapid test) varied, from 40% to 87%.^43^ Thus, for Ebola rapid tests with potential acceptable sensitivity, well designed clinical studies with larger sample sizes are necessary for an adequate assessment of their current performance.

Finally, we could not perform subgroup analysis comparing test performance by symptoms duration because of lack of data stratified by timing of the tests. Therefore, future field studies need to evaluate the performance of these tests in relation to symptoms duration. As an example, rapid diagnostic tests for severe acute respiratory syndrome coronavirus 2 are more sensitive (83.8%) when used within 7 days of symptoms onset than when used at later than 7 days (sensitivity of 61.5%).^44^ These limitations, however, would not modify the usefulness of Ebola rapid diagnostic tests as a tool for separating suspected Ebola patients while they are waiting for their RT–PCR results.

The strength of our study is that our literature was extensive, without any language restrictions, although only studies published in English and French were included. However, some studies were excluded because of low evidence on clinical performance, and studies could have been missed by our search strategy.

In conclusion, the results from this meta-analysis suggest that currently there is no commercial rapid diagnostic test for Ebola virus disease that has sufficient sensitivity and specificity to meet WHO standards. Despite the suboptimal performance, these tests still have clinical value because of their rapid turnaround time. Clinicians, especially those in settings where RT–PCR tests are not immediately available, should be aware of the existence, availability and limitations of the rapid tests. A negative result is unreliable in a subject highly suspected of having Ebola virus disease and the result must be confirmed using RT–PCR. Current commercially available tests include ReEBOV™, SD Zaire Ag, OraQuick, Ebola Antigen System and QuickNavi-Ebola,^45^ and health-care workers can procure rapid tests, such as OraQuick, from the US Centers for Disease Control and Prevention or WHO.^46^ Our findings stress a great need for more accurate rapid diagnostic tests for Ebola virus. Tests with improved performance should undergo additional field testing, if blood samples are available or conducted during future outbreaks.
